# Response to Letter regarding EpCAM-targeted near-infrared photoimmunotherapy (NIR-PIT) for the treatment of breast cancer

**DOI:** 10.1080/07853890.2025.2568984

**Published:** 2025-10-13

**Authors:** Aki Furusawa, Hisataka Kobayashi

**Affiliations:** Molecular Imaging Branch, Center for Cancer Research, National Cancer Institute, NIH, Bethesda, MD, USA

We appreciate the constructive comments provided by Mao et al. [1] regarding our study about EpCAM-targeted NIR-PIT[2]. I would like to address several key points raised.

The authors expressed concern regarding off-target cell loss in EpCAM-targeted NIR-PIT due to the Fc binding of the antibody used as AbPC. It is true that using full IgG as an NIR-PIT agent may result in some unavoidable damage to Fc receptor-expressing cells, such as NK cells. However, this loss is anticipated to be localized, with new cells migrating into the treatment site from nearby tissues, rendering the impact non-critical. We agree that a more comprehensive analysis of the effects on immune cells is essential for future research. Should it become necessary to prevent the loss of Fc-binding cells, theoretically, the use of F(ab’)2 (Fc region-removed IgG) could mitigate this issue. Nonetheless, considering the production costs and the shorter half-life of F(ab’)2, we believe its use may not be warranted in this context.

The authors also suggested that investigating intermediate-affinity anti-EpCAM antibodies could be beneficial, which is a valuable point. Our findings indicate that antibody clones with excessively high affinity can cause unintended side effects, whereas clones with a lower yet sufficient affinity might offer a safer alternative. We concur that intermediate-affinity antibodies may be optimal, and this avenue warrants further exploration.

Regarding the tumour models utilized in our study, we employed subcutaneous models to facilitate precise tracking of tumour size. Having demonstrated the efficacy of EpCAM-targeted NIR-PIT in killing cancer cells and inducing anticancer immunity, we are now planning to extend our investigations to orthotopic and metastatic models that better represent the complexity of patients’ cancers.

The authors raised concerns about delivering NIR light to internal organs, such as the colon, acknowledging the technical challenges involved. Several studies are underway to address these hurdles. For example, endoscopic devices have been tested in oral cancer models in mice [3]. Additionally, light-emitting fibre devices for limited light angles have been explored [4]. For larger tumours, fibre optic diffusers have been successfully used to deliver NIR light at depth in clinical settings. The image-guided interventions with optic diffusers are commonly employed for precise needle insertion [5]. As for real-time monitoring, the use of a device called Light Vision, which tracks IR700 fluorescence [6], has been investigated to monitor the fluorescent decay of IR700.

As the authors noted, NIR-PIT offers a personalized approach to cancer treatment by selecting targets based on the antigen expression profile of a patient’s tumour. Incorporating EpCAM into the repertoire of NIR-PIT targets could expand its applicability. Additionally, combining NIR-PIT with immune-modulating agents, tailored to the tumour’s immune microenvironment status, could further enhance the anticancer effect. Exploring agents beyond the anti-PD-1 used in our current study is a promising future direction.

The insights from our study, coupled with ongoing technological advancements, will undoubtedly contribute to the successful clinical application of EpCAM-targeted NIR-PIT.

## Data Availability

Data sharing is not applicable to this article, as no data was created or analysed in this article.
